# An Improved Test for Detecting Multiplicative Homeostatic Synaptic Scaling

**DOI:** 10.1371/journal.pone.0037364

**Published:** 2012-05-17

**Authors:** Jimok Kim, Richard W. Tsien, Bradley E. Alger

**Affiliations:** 1 Institute of Molecular Medicine and Genetics, Graduate Program in Neuroscience and Department of Neurology, Georgia Health Sciences University, Augusta, Georgia, United States of America; 2 Department of Molecular and Cellular Physiology, Stanford University School of Medicine, Stanford, California, United States of America; 3 Department of Physiology, Psychiatry and Program in Neuroscience, University of Maryland School of Medicine, Baltimore, Maryland, United States of America; Centre national de la recherche scientifique, University of Bordeaux, France

## Abstract

Homeostatic scaling of synaptic strengths is essential for maintenance of network “gain”, but also poses a risk of losing the distinctions among relative synaptic weights, which are possibly cellular correlates of memory storage. Multiplicative scaling of all synapses has been proposed as a mechanism that would preserve the relative weights among them, because they would all be proportionately adjusted. It is crucial for this hypothesis that all synapses be affected identically, but whether or not this actually occurs is difficult to determine directly. Mathematical tests for multiplicative synaptic scaling are presently carried out on distributions of miniature synaptic current amplitudes, but the accuracy of the test procedure has not been fully validated. We now show that the existence of an amplitude threshold for empirical detection of miniature synaptic currents limits the use of the most common method for detecting multiplicative changes. Our new method circumvents the problem by discarding the potentially distorting subthreshold values after computational scaling. This new method should be useful in assessing the underlying neurophysiological nature of a homeostatic synaptic scaling transformation, and therefore in evaluating its functional significance.

## Introduction

Hebbian synaptic plasticity, such as long-term potentiation, is considered a cellular correlate of memory [1], but also has the potential to destabilize a network as a result of its positive feedback regulation of synaptic efficacy [2], [3]. By offsetting extreme changes in activity by compensatory reductions, “synaptic homeostasis” [4], [5] has been proposed as a mechanism to stabilize neuronal circuits during accumulation of Hebbian plasticity [2], [3]. However, simple compensatory homeostatic regulation of synaptic strengths carries the risk of erasing the relative differences among synaptic weights that are inscribed by Hebbian plasticity, and that constitute the basis of information storage, i.e., memory. This problem could be avoided by a uniform multiplicative scaling process, in which strengths of all the synapses subject to the same level of activity perturbation were altered by the same factor, as this would leave the relative synaptic strengths unaltered [2], [3], [6]. Therefore, multiplicative scaling is a key concept that would enable homeostatic plasticity to achieve two beneficial functions simultaneously: network stabilization and memory preservation.

Because of the impracticality of assessing individually thousands of synapses in even a small neuronal circuit, a mathematical procedure has been developed to permit determination of whether or not multiplicative scaling occurred following a global alteration in neuronal activity. The outcome of this procedure is then used to constrain the kinds of underlying biophysical mechanisms of synaptic regulation within the network. For example, a possible mechanism for multiplicative synaptic scaling after global changes in neuronal activity, would be insertion or removal of AMPA receptors into/from spines by the same factor across all synapses [3], [4]. Accordingly, the accurate estimation of the scaling procedure will affect any conclusions about its functional relevance (see Discussion).

The concept of multiplicative scaling originally arose from a theoretical analysis of changes in the amplitudes of miniature excitatory postsynaptic currents (mEPSCs) following a period of global silencing or disinhibition of a network [6]. In this original study, the occurrence of multiplicative scaling was assessed by the degree of overlap between two distributions of mEPSC amplitudes when one distribution was scaled mathematically to match the other. If, after mathematical scaling, the distribution of mEPSC amplitudes obtained from activity-altered neurons overlapped with that of the control mEPSCs, it was concluded that all the excitatory synapses had been scaled multiplicatively [6].

Because the existence of true multiplicative scaling is critical for the conclusions that emerge from such studies, e.g., memory preservation, the validity of the test for scaling must be carefully examined. Inaccuracy in the determination of scaling patterns would challenge the suitability of the multiplicative scaling hypothesis to account for the data, and thereby potentially alter the biological interpretation of the synaptic scaling. We show here that limitations in the original scaling method can in fact lead to a distortion in the distributions of the mEPSCs, and accordingly could result in misleading conclusions. We now propose a new method that overcomes the problems, strengthens the test for multiplicative scaling, and thereby contributes to better interpretation of the empirical underpinnings of homeostatic synaptic plasticity and its functional significance.

## Results

### Conventional test for multiplicative scaling and its shortcoming

**Figure 1:**
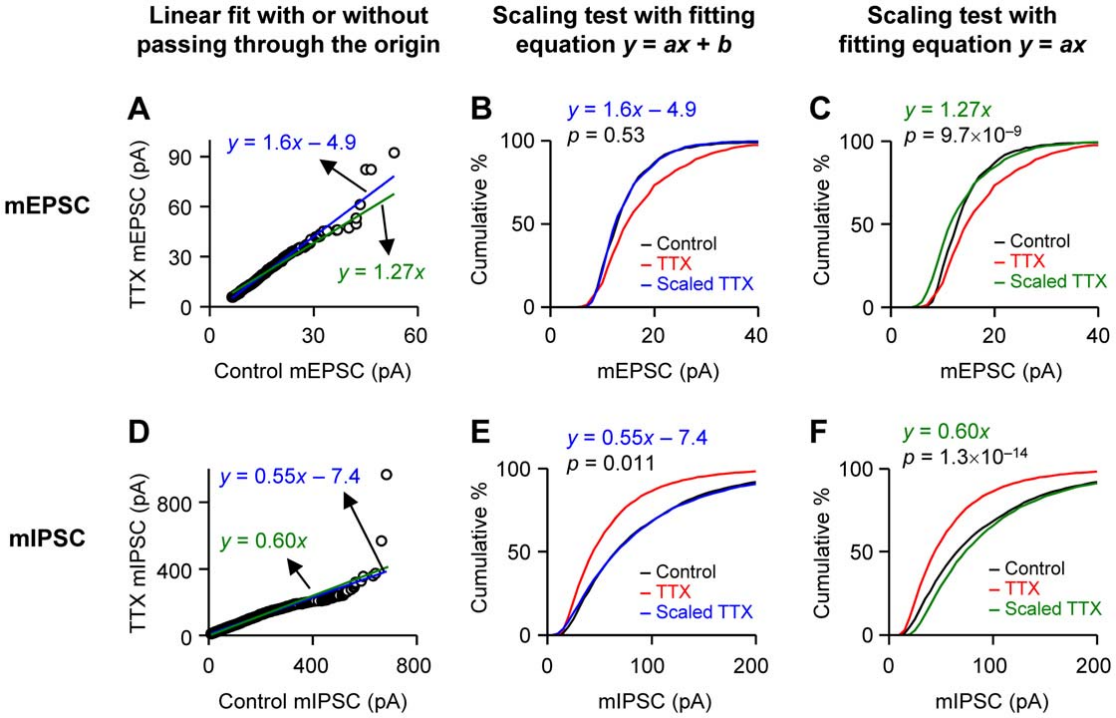
Test for multiplicative scaling of mEPSC amplitudes with a rank-order plot of amplitudes. **A.** mEPSCs were recorded from CA1 pyramidal neurons in slice cultures of rat hippocampus. For induction of homeostatic plasticity of synapses, slice cultures were treated with 1–1.5 µM TTX for 3–4 days. Rank-ordered amplitudes of mEPSCs in TTX-treated cells were plotted against rank-ordered control mEPSCs. A total of 700 mEPSCs, with 100 events per cell, were collected. The straight lines are linear fits with variable (blue) or zero (green) *y*-intercept. **B.** Cumulative histograms of mEPSC amplitudes. Individual mEPSC amplitudes in TTX-treated cells were transformed with the equation *y* = 1.6*x*–4.9. The distribution of transformed values almost exactly overlaps the control distribution. The *p* value is from a K-S test between control and scaled TTX group. **C.** Cumulative histograms were obtained as in B (control and TTX plots are the same as those in B), but the TTX group was scaled with the equation *y* = 1.27*x*. The *p* value from a K-S test shows significant differences between control and scaled TTX groups. **D.** mIPSCs were obtained from control and TTX-treated slice cultures of hippocampus. The rank-order plot (D) and the cumulative histograms (E,F) were constructed in a similar manner to those in A–C. **E.** Cumulative histogram of mIPSC amplitudes. The amplitudes of TTX-treated mIPSCs were transformed with *y* = 0.55*x*–7.4. Again, the transformed distribution shows nearly complete overlap with the control distribution. The *p* value is from a K-S test between control and scaled TTX group. **F.** The TTX group was scaled with the equation *y* = 0.60*x*. The distributions of the control and scaled TTX groups are significantly different from each other (K-S test).

To induce homeostatic scaling of excitatory synaptic transmission, we chronically blocked neuronal firing by treating slice cultures of rat hippocampus with 1–1.5 µM tetrodotoxin (TTX) for 3–4 days. As reported [7], the mean mEPSC amplitude of TTX-treated cells (17.4±0.77 pA; n = 7 cells) was larger than that of control cells (14.2±0.85 pA; n = 7) [*p*<10^−9^, Kolmogorov-Smirnov (K-S) test; Fig. 1A]. We employed a widely accepted method [6] to test for the occurrence of multiplicative scaling: first, mEPSC amplitudes of the TTX-treated cells were rank-ordered and plotted against the rank-ordered mEPSC amplitudes of the control cells. The rank was determined in each of control and TTX groups independently of the other group. Then the plot was fitted with a straight line to obtain the scaling function, *y* = 1.6*x*−4.9 (Fig. 1A). A small number of data points in the large-amplitude region (e.g., >40 pA) deviated from the straight line perhaps because of random variability in the number of receptors activated in some larger, unsaturated receptor patches. The number of these extreme values was quite low (<1% of the total) and the linear regression seemed to generate a reliable fitting of the majority of the data. After transforming individual mEPSC amplitudes of TTX-treated neurons with this equation, we constructed a cumulative frequency plot of the mEPSC amplitudes. The distribution of the scaled TTX data closely overlapped with that of control data (*p* = 0.53, K-S test; Fig. 1B). Conventionally, this result has been interpreted as meaning that multiplicative scaling of mEPSC amplitudes had occurred, but this conclusion actually does not follow, as discussed below.

Although it was originally proposed and is currently accepted that a successful transformation by *y* = *ax*+*b* can be considered multiplicative scaling [3], [6], this transformation in fact includes both multiplicative (*a*) and additive (*b*) components. In multiplicative scaling, all synapses should be scaled proportionately. In other words, the scaling factor (new synaptic strength/initial synaptic strength) must be the same for all the synapses. This, of course, occurs if the scaling equation is *y* = *ax*, where the scaling factor (*y*/*x*) is *a*, a constant. However, in the equation *y* = *ax*+*b*, the scaling factor is *a*+*b/x* and varies with initial synaptic strength, *x*. If synapses are scaled by *a*+*b/x*, stronger synapses (e.g., *x*»*b*) are scaled up to a lesser extent than weaker synapses (e.g., *x*≤*b*). The relationship among synaptic strengths would be distorted after this scaling process. The implications for the physiological data that are well fit by this function could be that the relative synaptic weightings within the population was not accurately preserved, and that information stored in the synaptic strengths was degraded or lost.

To determine if scaling carried out with *y* = *ax*+*b* reflected a significant deviation from the results expected from a purely multiplicative scaling, we used the transformation equation *y = ax* and compared the results. The rank-order plot was fitted with *y* = *ax* (Fig. 1A), and individual mEPSC amplitudes of TTX-treated cells were divided by *a*. When the TTX mEPSCs were scaled down by the slope of the fitted line, 1.27, the distribution of the scaled mEPSC amplitudes was significantly different from that of control mEPSCs (*p* = 9.7×10^−9^, K-S test; Fig. 1C). This was contrary to the conclusion reached when we fitted the data with *y = ax+b*, and suggested a failure of the simple multiplicative scaling hypothesis.

We obtained similar results with miniature inhibitory postsynaptic current (mIPSC) data. As expected for homeostatic scaling, the mean amplitude of mIPSCs in TTX-treated cells (58±6 pA; n = 7) was smaller than that of control mIPSCs (91±9 pA; n = 7; *p*<10^−64^, K-S test; Fig. 1D) [8]. The linear fit of the rank-ordered amplitudes was *y* = 0.55*x*−7.4 (Fig. 1D) and the distribution of the TTX data that were scaled by this equation was not significantly different from that of control data (*p* = 0.011, K-S test; Fig. 1E). Again, the conventional interpretation would be the scaling of activity-deprived mIPSCs was simply multiplicative [9]. We however further analyzed the mIPSC data using a pure multiplicative equation, *y* = 0.60*x*, which was obtained from a linear regression of the rank-ordered data (Fig. 1D). Similarly to the mEPSC data, the distribution of scaled mIPSC amplitudes obtained by dividing TTX mIPSCs by 0.60 was significantly different from the control distribution (*p* = 1.3×10^−14^, K-S test; Fig. 1F). These results and ideas suggest that the conventional test for multiplicative scaling (i.e., fitting rank-order plots with *y* = *ax*+*b*) has been misinterpreted, and the seemingly correct use of the test (i.e., fitting rank-order plots with *y* = *ax*) showed non-multiplicative scaling of our mE/IPSC data. This implies that the physiological changes of mE/IPSCs might encompass more complex processes than simple multiplicative scaling and instead involve sums of multiplicative and additive components.

### Source of the problems of the conventional test

**Figure 2:**
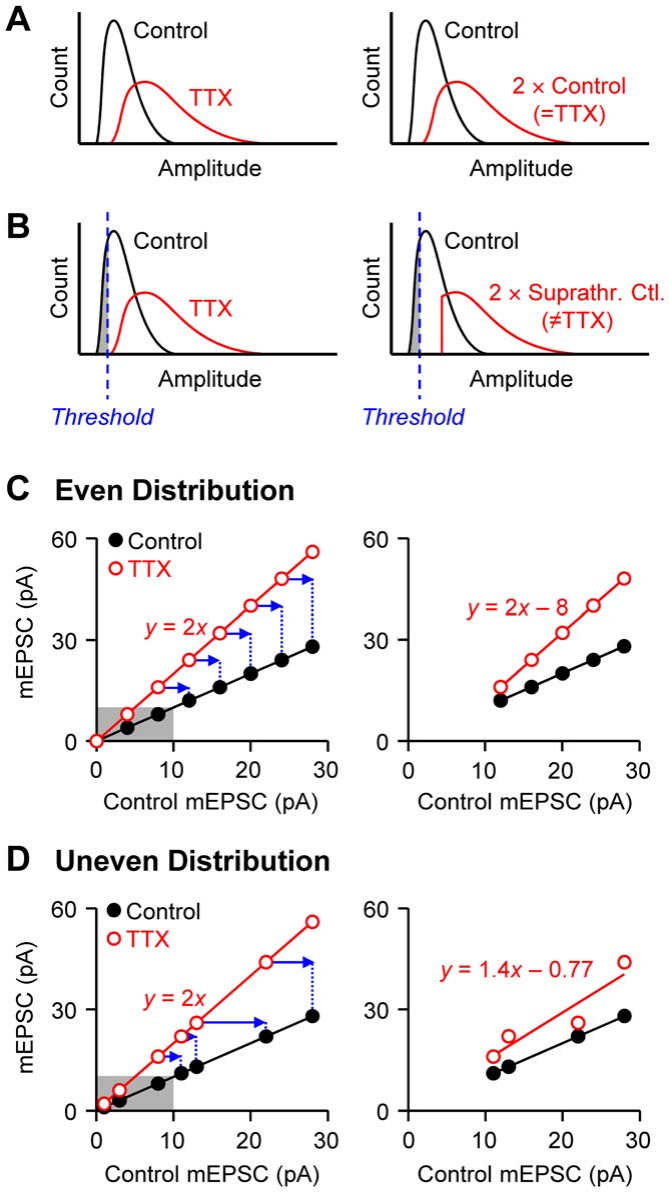
Schematic diagrams illustrating the inadequacy of the previous test for multiplicative scaling. **A.** Hypothetical distribution curves, in which all of the mEPSC amplitudes are included without a detection threshold. The TTX-treated mEPSCs are assumed to be two-fold larger than the controls (i.e., multiplicative scaling; left). If the control population is multiplied by two (right), the scaled control curve becomes identical to the TTX curve. **B.** Schematic diagrams similar to A, but a detection threshold of mEPSC amplitude limits the minimum amplitude (left), as in realistic mEPSC recordings. If the suprathreshold control values (i.e., excluding the gray area) are multiplied by two (right), the minimum offset of the scaled curve (red) will be shifted by two-fold, resulting in a mismatch with the TTX curve (left graph). This suggests that a transformation of *y = ax* cannot be used to test for multiplicative scaling when a detection threshold is present. **C.** Schematic diagrams show how the exclusion of subthreshold amplitudes invalidates the rank-order method of the conventional multiplicative scaling test. Hypothetical mEPSC amplitudes distributed evenly are rank-order plotted. TTX-treated mEPSCs (open circles) are assumed to be two-fold larger (*y* = 2*x*) than control ones (filled circles). The left graph shows an ideal, full range of mEPSC amplitudes, including the subthreshold values (<10 pA; gray area). If the subthreshold data are discarded, as in realistic mEPSC recordings, the rank-order plot of TTX data will be shifted (blue arrows). A linear fit of the TTX data in the resultant rank-order plot (right graph) now gives *y* = 2*x*–8. **D.** Schematic illustrations similar to C, but with data distributed unevenly. The TTX data (open circles) again constitute a multiplicative scaling of the controls by a two-fold (left graph). The exclusion of subthreshold data points (gray area) will cause a shift of the remaining data by different distances (blue arrows). A linear fit of the new rank-order plot of TTX-treated data (right graph) yields an equation that loses information about the original multiplicative scaling factor. When the TTX data in the right graph were fitted with a *y*-intercept of 0, the equation was *y* = 1.45*x* (line not shown), again different from the original scaling function.

However, before rejecting the hypothesis that multiplicative scaling can account for the data, we considered whether or not the conventional method of data fitting might have introduced errors that affected the results. The reason for the concern is the existence of an experimental detection threshold for mEPSC and mIPSC amplitudes, which can be problematical for the following reasons. First, when a fixed detection threshold limits the minimum amplitudes in control and treated populations, as in the case of experimental detection of mEPSCs, simple multiplication of one population must, in principle, always fail to overlap with the other population (compare Figs. 2A and B). This is because the smallest amplitudes in the control distribution will be below the detection level, but the synapses that give rise to them will nevertheless be boosted by the TTX treatment, and the smallest responses may then form part of the TTX-treated mEPSC population. Yet the mathematical multiplicative scaling process can only act on the suprathreshold responses, and will produce a distribution of which the cut-off level will also be shifted with respect to the minimum value of the experimental (e.g., TTX) group (Fig. 2B). As a result, the distributions of the TTX group (Fig. 2B left, red curve) and the scaled control group (Fig. 2B right, red curve) will not match each other. Only if there were no lower limit in a distribution, would *y = ax* suffice as an accurate test for multiplicative scaling in such cases (Fig. 2A).

Second, the rank-order plot method has limitations when non-detectable subthreshold values should be estimated from an extrapolation of suprathreshold data. In rank-order plots of experimental data, the smallest mEPSCs of control and TTX-treated cells are paired with each other, but the minimal TTX-treated mEPSCs may, again, be scaled up versions of subthreshold control mEPSCs, which would not have been detected experimentally (Figs. 2C,D). In an experimental rank-order plot, the smallest TTX mEPSC must be paired with the minimal control mEPSC, and therefore, the consequent rank-order plot of TTX-treated mEPSCs represents a shift of an ideal rank-order plot that includes subthreshold amplitudes (Fig. 2C). If the data points are evenly distributed across various amplitudes (Fig. 2C), the slope of the original linear fit will be preserved in the plot of suprathreshold data. In this case, the slope alone (without the *y*-intercept) might appear to be an accurate scaling factor [10]. Amplitudes of synaptic currents, however, typically distribute unevenly [11], [12], [13] (cf. Figs. 1A,D). If subthreshold points are excluded from unevenly distributed data, the remaining data points in a rank-order plot are shifted by heterogeneous distances (Fig. 2D). In this case, the calculated slope, i.e., the scaling factor, diverges from its true value. Consequently, a simple linear fit to the suprathreshold data will not reveal the information present in the original scaling function. In sum, the existence of a detection threshold for mE/IPSC amplitudes challenges the validity of conclusions based on the conventional test for multiplicative scaling.

### Alternative method for testing multiplicative scaling

**Figure 3:**
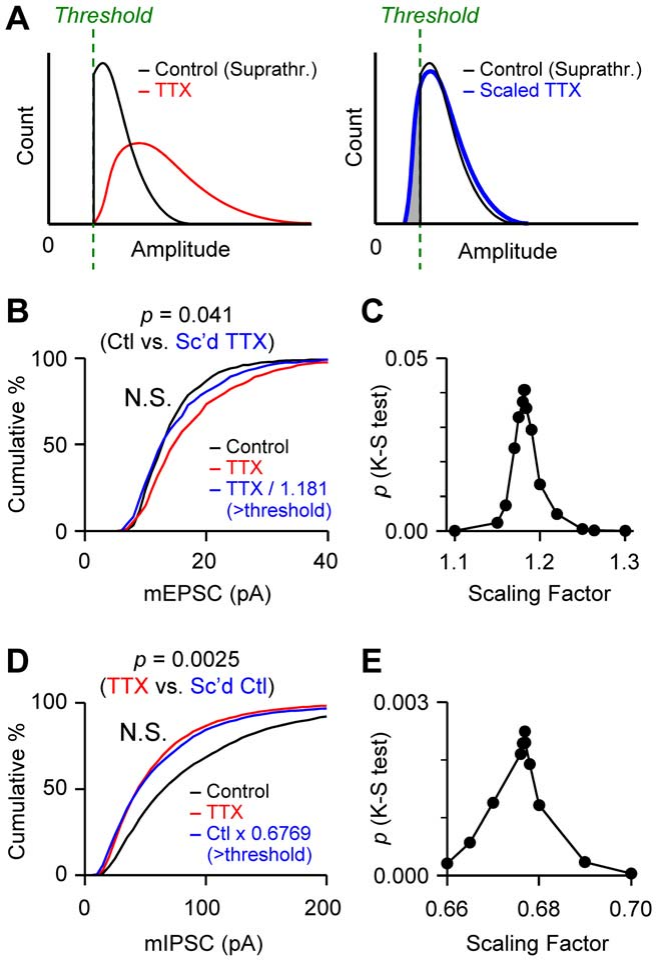
A new method of testing for the occurrence of multiplicative scaling. **A.** Schematic diagrams of distributions of mEPSC amplitudes illustrate the new method of estimating a true multiplicative scaling factor. Only suprathreshold data (Suprathr.) are shown in the left graph. Multiplicative down-scaling of TTX data (from red curve to blue one) results in a small portion of data (gray) falling below the detection threshold. Because the data in the gray area are invisible in experimental recording, they are discarded for comparison with the control data. The degree of overlap is measured between the control (black curve) and the scaled TTX data (blue curve) excluding the gray area. **B.** Cumulative histograms of experimental data shown in Fig. 1A. To derive the distribution of scaled TTX data (blue), the TTX-treated mEPSCs were divided by 1.181, which yielded the maximum overlap between control and scaled TTX data excluding subthreshold values. The threshold is defined as the smallest detectable amplitude of control mEPSCs. A K-S test showed no significant difference between control and scaled TTX groups (*p*>10^−4^), suggesting the occurrence of multiplicative scaling. **C.** With other scaling factors, the degrees of overlap between control and scaled TTX data excluding subthreshold values were also examined. The degree of overlap of the histograms was quantified by confidence level (*p* value) of K-S test. The largest *p* (0.041) was obtained when mEPSC amplitudes of the TTX group were divided by 1.181. **D.** A test for multiplicative scaling was performed with mIPSCs in a similar manner to that in A–C. Because the mean amplitude of control mIPSCs was larger than that of TTX-treated ones, control mIPSC amplitudes were scaled down by multiplying by 0.6769. A K-S test between scaled control and TTX data (*p*>10^−4^) revealed multiplicative scaling. **E.** The degrees of overlap between scaled control and TTX data excluding subthreshold values were assessed with various scaling factors. The largest *p* (0.0025) was obtained with a scaling factor of 0.6769.

In general, if one population is derived from another by multiplicative scaling up, then this should be a reversible operation; i.e., scaling the experimental distribution down by an appropriate factor should also produce overlap between experimental and control distributions. However, the existence of the detection threshold can create a problem here as well. For example, if an experimental population with a larger mean amplitude, e.g., mEPSCs of TTX-treated cells, is scaled down to determine its overlap with a control distribution, the low-end mEPSCs will fall below the actual experimental detection threshold (Fig. 3A). Because detectable mEPSCs of control cells cannot have such small amplitudes, the calculated, scaled- down TTX mEPSCs that fall below the threshold will not have control counterparts. This results in a non-overlapping portion of the down-scaled distribution (gray area in Fig. 3A).

Noting that this gray region should be similar to the subthreshold region in the original control distribution (cf. Figs. 2B and 3A), we are led to propose another way to determine the degree of overlap of two distributions acquired in the presence of a detection threshold. In this method, we exclude the down-scaled TTX mEPSCs that fall below the detection threshold, defined as the smallest mEPSC amplitude in the control group. We then determine the scaling factor that will provide the best overlap between the control mEPSCs and the suprathreshold mEPSCs in the down-scaled TTX group. With the experimental data shown in Fig. 1A, we first scaled down the individual mEPSCs of TTX-treated cells with an arbitrary scaling factor, and then excluded the scaled TTX amplitudes that fell below the threshold. The distribution of the suprathreshold TTX data was then compared with that of control data, and the degree of overlap was judged with a K-S test. This procedure was repeated with a series of scaling factors, and the number that produced the best overlap (i.e., the highest *p* value of the K-S test) was chosen as the factor to be used for multiplicative scaling (Figs. 3B,C). In this example, when mEPSC amplitudes of TTX-treated cells were scaled down by a factor of 1.181, the scaled TTX population overlapped most extensively with the control distribution (Figs. 3B,C). This was therefore the best estimate of the underlying multiplicative scaling factor. Using this factor, we find that the TTX-treated mEPSCs do scale in a true multiplicative way with the original data (*p* = 0.041, K-S test, i.e. not significant, see Materials and Methods; Fig. 3B). Thus, by taking into consideration the occurrence of subthreshold data, our new method reveals that, in contrast to the conclusion obtained with the conventional method (e.g., Fig. 1C), a simple *y* = *ax* transformation adequately represents the underlying scaling process – it was not necessary to include an additive correction factor.

The scaling pattern of mIPSCs was also tested with the new method. The control mIPSCs, which had a larger mean amplitude than did the TTX group, were scaled down by dividing the amplitudes by various scaling factors. The down-scaled control data that fell below the detection threshold were discarded, and then the scaled control mIPSCs above the threshold were compared with the TTX-treated mIPSCs. The best overlap between the scaled control and the TTX groups was generated with a scaling factor of 0.6769 (*p* = 0.0025, K-S test; Figs. 3D,E). This result again shows no significant difference between the two distributions and therefore implies a multiplicative scaling of mIPSCs. Thus our procedure may be adequate, unlike the method of rank-order plots, for assessing multiplicative scaling when a detection threshold affects data collection. As a different alternative approach, we also tried to determine if two mEPSC distributions match by comparing their means, but found this to be less sensitive in determining the maximum overlap of two distributions (Fig. S1). The previous test for multiplicative scaling and ours made two contrasting conclusions (Table 1): the original method implied that transformations involving mixtures of multiplicative and additive scaling factors were in operation for both mEPSCs and mIPSC, whereas our test was able to extract purely multiplicative changes.

**Table 1:** Summary of *p* values from K-S tests between two populations of mEPSC or mIPSC amplitudes.

	Previous method of rank-ordering (Figs. 1B,E)	Previous method of rank-ordering (Figs. 1C,F)	New method (Figs. 3B,D)
Rank-order fitting equation	*y = ax+b*	*y = ax*	N.A.
K-S test of experimental mEPSC data	0.53 (N.S.)	9.7×10^−9^ (*)	0.041 (N.S.)
Interpretation of the mEPSC scaling	multiplicative & additive	not multiplicative	Multiplicative
K-S test of experimental mIPSC data	0.011 (N.S.)	1.3×10^−14^ (*)	0.0025 (N.S.)
Interpretation of the mIPSC scaling	multiplicative & additive	not multiplicative	Multiplicative

Amplitudes of TTX-treated mE/IPSCs (Figs. 1 and 3B) or control mIPSCs (Fig. 3D) were scaled with the indicated scaling equations. For both mEPSCs and mIPSCs, the widely accepted method of rank-order plotting suggested a mixture of multiplicative and additive changes, but the new test proposed here revealed multiplicative scaling only. N.A., not applicable. N.S., not significant.

* , significant, *p*<10^−4^.

### Comparison between the two methods

**Figure 4:**
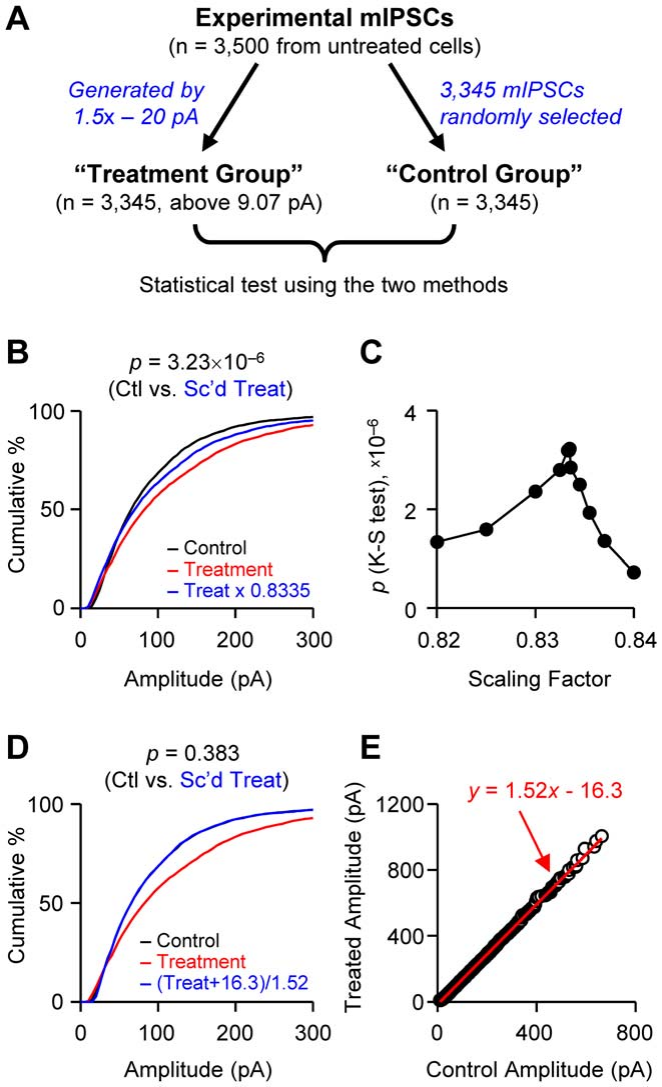
Tests for multiplicative scaling using two different methods and artificially generated data sets. **A.** Data generation scheme. mIPSC amplitudes (n = 3,500) recorded experimentally from untreated cells were arithmetically transformed: i.e., they were multiplied by 1.5 and subtracted by 20 pA. After the transformation, 3,345 values were above the detection threshold and this set was called the “treatment group”. Randomly selected values from the experimental group made up the “control group” (n = 3,345). **B–C.** As in Fig. 3, the treatment group was scaled down and compared with the control group using K-S tests. When the scaling factor was 0.8335, the distribution of the scaled treated group most completely overlapped that of the control group. The *p* value (K-S test) shows significant difference between the control and scaled treatment groups. **D–E.** In the conventional test, the amplitudes of control and treatment groups were rank-ordered and plotted against each other. The treatment group was scaled back using the linear regression equation. The distributions of control and scaled treatment groups were not significantly different from each other (K-S test).

We examined the sensitivity of our method with artificially generated data, to determine if it could correctly identify a non-multiplicative scaling transformation. To generate a data set that is scaled in a non-multiplicative fashion, we arithmetically transformed mIPSC amplitudes recorded experimentally from untreated cells (Fig. 4A). Each one of 3,500 amplitude values was multiplied by 1.5 and subtracted by 20 pA to generate a scaling process with both multiplicative and subtractive components. This transformation resulted in 3,345 of 3,500 amplitude values that remained above the detection threshold, which was the smallest amplitude in real experiments, 9.07 pA. This subset of data was called the “treatment group”. The “control group” should be in principle the mother group containing all 3,500 values, but for the data pairing in a rank-order plot, we needed to select the same number (3,345) of data points. The control group therefore was made by randomly selecting 3,345 values from the mother group. The random selection kept the distribution property of the control group the same as that of the mother group (*p* = 1, K-S test between control and mother groups; data not shown).

If our new method is sufficiently sensitive, it should be able to detect the non-multiplicative scaling of the treatment group when compared with the control group. Using the test introduced in Fig. 3, we scaled down the treatment group and compared it with the control group using a K-S test (Fig. 4B). When the scaling factor was 0.8335, the distribution of the scaled treatment group showed the most overlap with that of the control group (i.e., the highest *p* value of K-S test; Figs. 4B,C). However, even when the overlap was maximized, the two distributions were significantly different from each other (*p* = 3.23×10^−6^, K-S test), suggesting that the treatment group was not the result of multiplicative scaling of the control group. This shows that our test methods can effectively detect a non-multiplicative scaling transformation.

The test results arising from the conventional method (Figs. 4D,E) are in clear contrast to that of our new test. For the conventional test, we rank-ordered the control and treatment groups respectively and plotted the rank-ordered treatment values against the control ones. Linear regression of the rank-ordered data generated a fitting line of *y* = 1.52*x*−16.3 (Fig. 4E). The treatment group was scaled back using this equation and compared with the control group. The distributions of the control and scaled treatment groups were not significantly different from each other (*p* = 0.383, K-S test; Fig. 4D), thus giving the impression that the treatment group might be scaled multiplicatively from the control data. We additionally examined the data using the conventional test with origin-passing linear regression, *y* = 1.420*x*, but the conclusion was the same: the scaled treatment group had a similar distribution with that of the control group (*p* = 0.113, K-S test; data not shown). These results indicate once again that the conventional test can lead to false conclusions about underlying scaling transformations.

## Discussion

We have re-examined the original procedure that was developed to assess the pattern of homeostatic synaptic plasticity, which can occur during development or disorders of neural networks. For example, the conventional method appeared to show that a population of synapses underwent a multiplicative scaling transformation during the homeostatic plasticity [6]. This procedure is based on comparison of amplitude distributions, but the existence of detection thresholds led to the requirement of the correction factor. As a result, the scaling pattern was determined to be a transformation of *y* = *ax*+*b*, rather than simply *y* = *ax*. While this small factor (*b*) was thought to be negligible, we find that in fact it markedly altered the analysis and yielded inaccurate results. To overcome this problem, we developed a new method, in which subthreshold values that can distort the test are discarded before statistical comparisons are made. The new method not only produces more accurate assessments of the existence of multiplicative scaling, but it also successfully detects the occurrence of a non-multiplicative scaling transformation as shown with the artificially generated data (Fig. 4). The net result is a more sensitive test that can distinguish multiplicative scaling from other transformations. Some physiological implications of these results, comparison of the new method with other methods, and limitations of the new method are discussed below.

The question of whether or not multiplicative scaling accurately describes homeostatic plasticity within a synaptic population has important consequences for understanding the nature of the plasticity. No test that examines a population of events can unambiguously identify the underlying cellular mechanisms involved at each synapse. Nevertheless, a truly multiplicative transformation would imply that: 1) every synapse in the population was affected and, 2) every synapse was affected to a degree proportional to its own original strength. Hence, whether or not a transformation of population responses is truly multiplicative may be useful in *excluding* certain potential candidate mechanisms. For example, if multiplicative scaling accurately describes enhancement of synaptic strengths across a population, then any form of plasticity that affected only a subset of synapses would be ruled out as being solely responsible. Well-established candidates such as: 1) the unsilencing of silent synapses, 2) the selective elimination of only weak synapses, 3) the emergence of new synapses, or 4) addition of a fixed number (rather than percentage) of receptors to each synapse, are incompatible with the observation of a multiplicative scaling of a population of events. Similarly, if the population showed a multiplicative down-scaling, then selective elimination of a subset of synapses, or the removal of a fixed number of receptors from each synapse, could be ruled out. In contrast, rejection of the multiplicative scaling hypothesis might mean that the population was not uniformly altered, and this could lead to a search for the relevant groups of synapses and investigation of the mechanism working at each subset. In this case, the kinds of mechanisms that are incompatible with multiplicative scaling would now be favored. This consideration underscores the importance of correctly determining whether or not multiplicative scaling actually occurs.

There have been other variations [8], [10], [14] of the original analysis method [6]. In one study [10], rank-order plots of mE/IPSCs were fitted with *y* = *ax*+*b*, but only the slope was used as a scaling factor with the aim of obtaining a pure multiplicative transformation. However, this cannot overcome the problem of subthreshold values, as explained in Fig. 2D. In another study [14], control mEPSCs were multiplied by a factor without additive correction to match the “average” mEPSC amplitudes of control and treatment groups. However, matching of mean amplitudes does not guarantee the maximum overlap of two distribution curves (Fig. S1). Alternatively, linear fitting of a rank-order plot was done on only 10–90th percentile of mIPSC amplitudes to avoid distortion of fitting result caused by near-threshold values and large, scarce events [8]. Because this study again used *y* = *ax*+*b*, it could not conclusively identify true multiplicative scaling. Previous studies [10], [14] determined, using modified traditional test methods, that the homeostatic plasticity of mEPSCs in vivo reflected non-multiplicative scaling. It would be informative if these data were re-analyzed using the new test.

Despite its advantages, our new method does have some limitations. Because subthreshold values after arithmetic transformation are discarded, very small amplitudes are not subjected to the test. Therefore, the scaling pattern of very small events might not always be reliably determined. To investigate this possibility, we observed that the data generated by *ax*+*b* (Fig. 4) indeed had different scaling rules for small and large amplitude events, and that our method successfully detected the non-multiplicative scaling transformation. However, we cannot conclude from this single example that the new method will always succeed, and the caveat must be kept in mind. It would be interesting to examine multiplicative scaling in cells that have very large miniature synaptic currents. If all large mE/IPSCs could be accurately detected, a test for multiplicative scaling should not require the threshold-related correction that is introduced here.

It is of course possible to imagine more complicated forms of non-multiplicative scaling that would not be detected by our analytical procedure (or others). For example, if subsets of synapses within a population experienced opposite and off-setting changes (one group became stronger and another weaker in a precisely balanced way), this might erroneously appear to be a multiplicative scaling effect. An ideal way to remove such limitations would involve specifically tracking each of thousands of individual synapses over the time course of several days or more. Such an approach is presently beyond the reach of current experimental methods, and until it is, analytical methods for assessing population changes, as proposed here, should continue to be useful.

The maintenance of relative synaptic weights, i.e., memory stored at synapses, could be a challenging task for neurons during homeostatic synaptic plasticity. It is believed that the risk of memory loss during homeostatic scaling could be prevented by global and multiplicative scaling [2], [3]. This model contains two slightly different concepts: first, “global” scaling means that neurons can sense their own firing rate and adjust the strengths of all synapse onto them in a cell-autonomous manner [4], [15]. Second, multiplicative scaling implies the magnitude of change (i.e., fold change) is maintained across synapses [2], [3]. The evidence for global scaling is undoubtedly strong [16], but some observations are contradictory to the global scaling hypothesis and support the “local scaling” hypothesis [17], [18], [19], [20]. In the local scaling of synapses, an individual synapse has its own target strength and any deviation from it would be adjusted homeostatically [4], [15]. The local homeostatic scaling appears to be an intrinsic eraser of Hebbian plasticity, but computational studies suggested that relative synaptic strengths can still be preserved [21], [22], for example, by working as dendrite-wide scaling [22], [23], [24].

Unlike the global scaling, the existence of multiplicative scaling has weaker experimental support. The determination of the occurrence of multiplicative scaling has entirely depended on the test using rank-order plots of miniature synaptic current [6]. We have stressed here the major problems of the conventional method. A transformation of amplitudes by *y* = *ax*+*b* should not be interpreted as multiplicative scaling but instead a sum of multiplicative and additive changes. In fact, this type of transformation simply means that two populations are on the same distribution function, i.e., of the same shape. For such distributions, multiplication of one population and then addition of a constant (i.e., sliding of distribution histogram on *x*-axis) will always result in overlap with the other distribution. Therefore, overlap after transformation by *y = ax+b* can be used as an indication that two populations have the same distribution function (for example, an alpha function), or the same skewness of the distributions as tested with the artificially generated data (Figs. 4D,E). Even a fitting of rank-order plots with *y* = *ax* cannot reveal the underlying scaling pattern because of subthreshold values (Figs. 2C,D) and this is confirmed by artificially generated data (Fig. 4).

Despite the multiplicative synaptic scaling that was revealed by our new test, recent experimental findings conflict with the hypothesis that simple multiplicative scaling always occurs. Homeostatic plasticity onto a given neuron is afferent- or target-specific, resulting in heterogeneous changes [7], [8], [25], [26], [27], [28]. Non-uniform homeostatic plasticity also occurs even with purely excitatory synapses in neuronal cultures, where some subsets of synapses are affected more than others [29], [30]. These examples of heterogeneous plasticity are different from the aforementioned local synaptic scaling because these heterogeneities occur even when all the synapses experience the same manipulation, e.g., activity deprivation, whereas the local scaling postulates any synapses of which activity is perturbed would change its strength. However, multiplicative scaling and synapse-specific homeostatic plasticity are not necessarily mutually exclusive if multiplicative scaling occurs uniformly across one type of synapse. In addition, if homeostatic scaling (i.e., amplitude change) originates from a postsynaptic mechanism and other afferent-specific modifications are presynaptic, multiplicative scaling may be co-expressed with heterogeneous, synapses-specific changes. Indeed, this appears to be the case for inhibitory synapses in the hippocampus: mIPSCs in TTX-treated slice cultures display multiplicative scaling (Fig. 3D), whereas GABAergic synapses in the same preparation experience synapse-specific changes in presynaptic release probability [8].

Our analysis revealed seemingly authentic multiplicative scaling of mEPSCs and mIPSCs in activity-deprived hippocampal slice cultures. Despite the beneficial roles of homeostatic plasticity in network maintenance and memory preservation, it may also have adverse effects. Homeostatic increases in excitability might elevate the chance of epileptic activity, or network destabilization [7], [31], [32]. These studies suggest that homeostatic plasticity might have both good and bad influences on network operation. The detailed evaluation of functional significance of synaptic homeostasis is critical in understanding the adaptation of neuronal networks to chronic activity perturbation, and the determination of synaptic scaling patterns will be an essential component of this endeavor.

## Materials and Methods

### Preparation of slice cultures

The raw data for this analysis were obtained during previous investigations of mEPSCs [7] and mIPSCs [8]. As reported [7], hippocampal organotypic slice cultures were made from 6–7-day-old Sprague-Dawley rats (Charles-River Laboratories, Wilmington, MA). Slices were cut in a saline consisting of (in mM) 122 NaCl, 3 KCl, 26 NaHCO_3_, 1 NaH_2_PO_4_, 2 CaCl_2_, 2 MgCl_2_, 20 glucose (300–305 mOsm; bubbled with 95% O_2_/5% CO_2_), and cultured on membrane (Millipore, Billerica, MA) at the interface of air with 5% CO_2_ and culture medium at 37°C. Slice cultures used for mIPSC recordings were made similarly from hippocampi of 15-day-old male rats [8]. The protocols were approved by the Institutional Animal Care and Use Committee of Stanford University or of the University of Maryland (protocol number 0609001).

### Electrophysiology

Slice cultures of age 21–25 days in vitro (for mEPSC) or 18–22 days in vitro (for mIPSC) were used for electrophysiology. Synaptic currents were recorded from CA1 pyramidal neurons with a whole-cell voltage clamp technique at 22–24°C. Electrode resistance (3–4 MΩ) and series resistance (<25 MΩ) were stable within 15% during the experiments. Data were collected using Axopatch 1C or Axopatch 200B (Molecular Devices, Sunnyvale, CA), filtered at 1 or 2 kHz and digitized at 20 or 50 kHz in Clampex 9 program (Molecular Devices). mEPSCs were recorded at −70 mV in the normal saline containing TTX (1 µM), gabazine (10 µM), and d-AP5 (50 µM). The pipette solution contained (in mM) 120 CsCH_3_SO_3_, 8 CsCl, 1 MgCl_2_, 0.4 EGTA, 2 ATP-Mg, 0.3 GTP-tris, 10 HEPES, 5 QX314 bromide and 10 phosphocreatine (pH 7.20 with CsOH, 295 mOsm). The perfusion rate of bath solution was ∼1.5 ml/min. mIPSCs were recorded with KCl-based pipette solution and the details were described previously [8].

### Data Analysis

Individual mE/IPSCs were detected with the Clampfit program (Axon Instruments) with a template made from our own data. To ensure that each cell contributed equally to statistical analysis, we used 100 mEPSCs or 500 mIPSCs per cell. For statistics, all of the mE/IPSC amplitudes were pooled and subjected to a K-S test with the PAST program (http://folk.uio.no/ohammer/past/). mE/IPSCs were inward currents, but the signs of amplitudes were converted to positive values for computational convenience. The amplitude of the smallest mEPSC in the control group, 6.43 pA, was used as a detection threshold when subthreshold values were excluded from the down-scaled mEPSC amplitudes of the TTX group in Figs. 3B,C. For mIPSCs, the detection threshold in Figs. 3D,E was defined as the smallest mIPSC amplitude in the TTX-treated group, 9.42 pA. All data represent mean ± SEM. We used a stringent confidence level of K-S tests, *p*<10^−4^, because the sample sizes of the mE/IPSC data sets are large.

## Supporting Information

Figure S1 **A match of two distribution curves is determined from the means of mE/IPSC amplitudes.** **A.** Cumulative histograms of experimentally recorded mEPSCs that were presented in Fig. 1A. The method of data processing (scaling and exclusion of subthreshold values) was the same as that in Fig. 3, except that the best match of control and scaled TTX distributions was determined by the similarity of the means of the two groups. The scaled TTX data (blue) were generated by dividing the TTX-treated mEPSCs by 1.264. The threshold was defined as the smallest amplitude of control mEPSCs. K-S test showed a similarity between control and scaled TTX data (*p*>10^−4^) suggesting an occurrence of multiplicative scaling, but the *p* value is much smaller than that in Fig. 3B, suggesting that this method is less sensitive than the test in Fig. 3 in determining the maximum overlap of two populations. **B.** With other scaling factors, the mean mEPSC amplitude of the scaled TTX group was compared with that of the control group, after the exclusion of subthreshold values. Two mean values were the same with a scaling factor of 1.264. **C–D.** The same process as in A–B was done with the mIPSC data. When the control mIPSC amplitudes were multiplied by 0.6295 and subthreshold values were discarded, the mean amplitude of the scaled control data was the same as the mean of TTX-treated data. The distributions of the TTX and scaled control groups were significantly different from each other (*p* = 1.95×10^−7^, K-S test), suggesting a lack of multiplicative scaling of mIPSCs.
(TIF)
