# Supplementary figures and images for: An Improved Test for Detecting Multiplicative Homeostatic Synaptic Scaling
Source: PLoS One. 2012 May 17;7(5):e37364. doi: 10.1371/journal.pone.0037364 (PMC3355135; doi:10.1371/journal.pone.0037364)

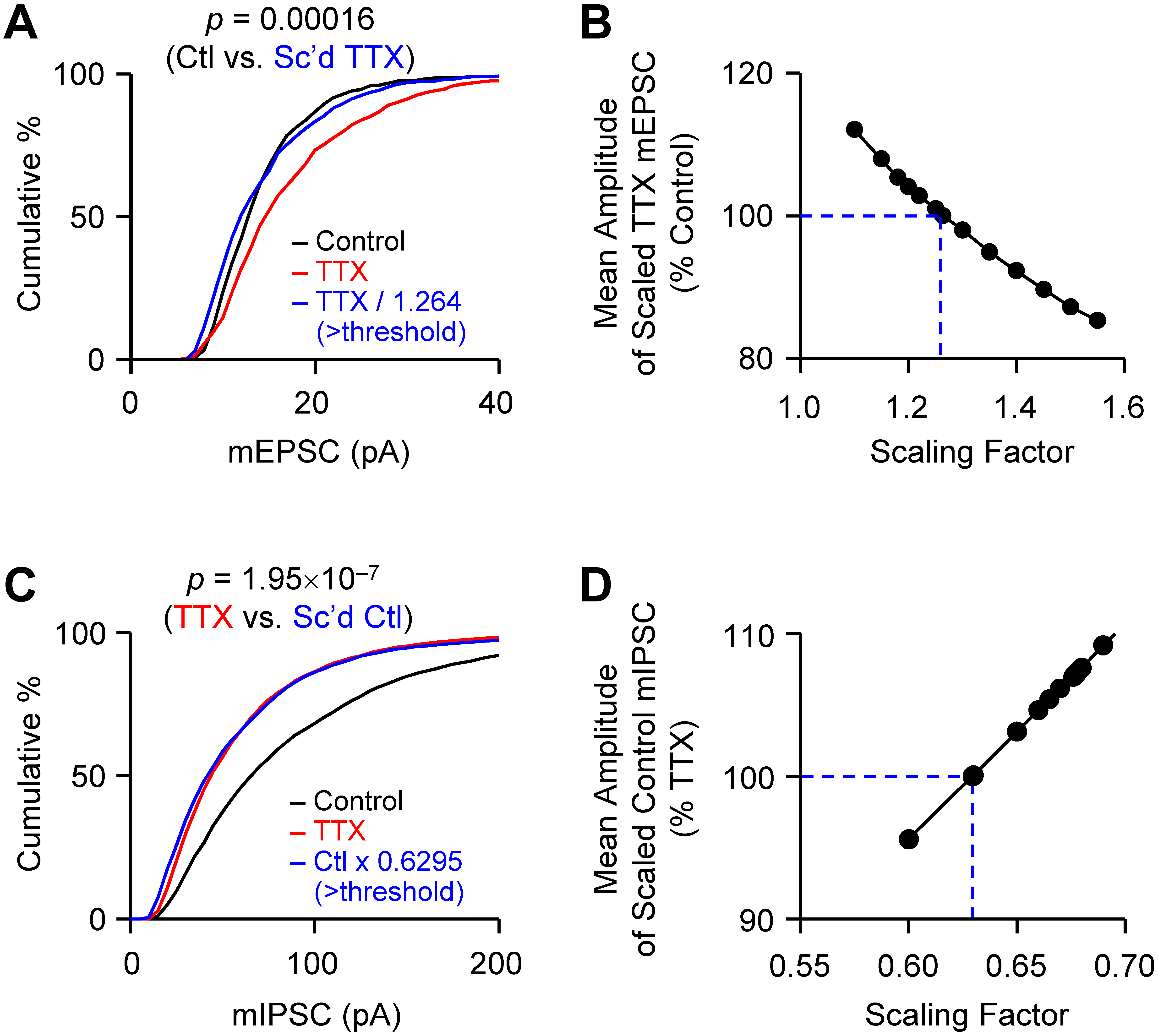

Supplement: Figure S1 — A match of two distribution curves is determined from the means of mE/IPSC amplitudes. A. Cumulative histograms of experimentally recorded mEPSCs that were presented in Fig. 1A. The method of data processing (scaling and exclusion of subthreshold values) was the same as that in Fig. 3, except that the best match of control and scaled TTX distributions was determined by the similarity of the means of the two groups. The scaled TTX data (blue) were generated by dividing the TTX-treated mEPSCs by 1.264. The threshold was defined as the smallest amplitude of control mEPSCs. K-S test showed a similarity between control and scaled TTX data (p>10−4) suggesting an occurrence of multiplicative scaling, but the p value is much smaller than that in Fig. 3B, suggesting that this method is less sensitive than the test in Fig. 3 in determining the maximum overlap of two populations. B. With other scaling factors, the mean mEPSC amplitude of the scaled TTX group was compared with that of the control group, after the exclusion of subthreshold values. Two mean values were the same with a scaling factor of 1.264. C–D. The same process as in A–B was done with the mIPSC data. When the control mIPSC amplitudes were multiplied by 0.6295 and subthreshold values were discarded, the mean amplitude of the scaled control data was the same as the mean of TTX-treated data. The distributions of the TTX and scaled control groups were significantly different from each other (p = 1.95×10^−7^, K-S test), suggesting a lack of multiplicative scaling of mIPSCs. (TIF) [file pone.0037364.s001.tif]
